# Functional enhancement of exosomes derived from NK cells by IL-15 and IL-21 synergy against hepatocellular carcinoma cells: The cytotoxicity and apoptosis in vitro study

**DOI:** 10.1016/j.heliyon.2023.e16962

**Published:** 2023-06-02

**Authors:** In-Young Kim, Ho Yong Kim, Hyeong-woo Song, Jong-Oh Park, You Hee Choi, Eunpyo Choi

**Affiliations:** a Korea Institute of Medical Microrobotics, 43-26 Cheomdangwagi-ro, Buk-gu, Gwangju 61011, Republic of Korea; b School of Mechanical Engineering, Chonnam National University, 77 Yongbong-ro, Buk-gu, Gwangju 61186, Republic of Korea

**Keywords:** Apoptosis, Cytokines, Cytotoxicity, Exosomes, Hepatocellular carcinoma, Natural killer cells

## Abstract

Exosomes are released by various cells, including natural killer (NK) cells and transport signaling molecules for the intercellular communication. Hepatocellular carcinoma (HCC), also known as primary liver cancer, is often inoperable and difficult to accurate diagnosis. Notably, the prognosis and underlying mechanisms of HCC are not fully understood. Exosomes-derived NK cells (NK-exos) express unique cytotoxic proteins with a killing ability in tumors and can easily penetrate tumor tissues to improve their targeting ability. NK cell functions, inducing cellular cytotoxicity are modulated by cytokines such as interleukin (IL)-15 and IL-21. However, the mechanisms and effects of cytokines-stimulated NK-exos for the treatment of liver cancer, including HCC, are not well known. In this study, we aimed to investigate the synergistic anti-tumor effects of NK-exos stimulated with IL-15 and IL-21 (NK-exos^IL−15/21^) in Hep3B cells. Our findings revealed that NK-exos^IL−15/21^ expressed cytotoxic proteins (perforin and granzyme B) and contained typical exosome markers (CD9 and CD63) within the size range of 100–150 nm. Moreover, we demonstrated that NK-exos^IL−15/21^ induced the enhancement of cytotoxicity and apoptotic activity in Hep3B cells by activating the specific pro-apoptotic proteins (Bax, cleaved caspase 3, cleaved PARP, perforin, and granzyme B) and inhibiting the anti-apoptotic protein (Bcl-2). In summary, our results suggest that NK-exos^IL−15/21^ regulate strong anti-tumor effects of HCC cells, by increasing the cytotoxicity and apoptosis through the activation of specific cytotoxic molecules.

## 1 Introduction

Hepatocellular carcinoma (HCC), also known as primary liver cancer, is one of the top five leading causes of cancer death [1,2]. The surgery and liver transplantation offer potential cures for early-stage HCC, the recurrence rate after curative treatment exceeds 70% [3]. Even in cases when surgery is performed on early-stage HCC, the survival rate is unsatisfactory. Diagnosis of HCC occurs frequently at an advanced stage, rendering many patients ineligible for therapies. Additionally, conventional systemic chemotherapy has shown minimal survival benefits and limited efficacy [4]. The overall prognosis and precise underlying mechanisms of HCC are not yet to be defined. Hence, novel strategies based on cancer immunotherapy are urgently needed for the therapy of liver cancer, including HCC.

Natural killer (NK) cells are the essential regulator in immune system [5]. They modulate the apoptosis in target cells by releasing apoptotic proteins (e.g., perforin and granzyme B) and by binding death receptor ligands (e.g., Fas ligand [FasL] and tumor necrosis factor [TNF]-related apoptosis-inducing ligand [TRAIL]) with their specific receptors [6,7]. NK cells can also be exhibited the functional effects through cytokine-mediated cytotoxicity [8]. In cancer patients, various cytokines have demonstrated cooperative effects of NK cells. IL-12, IL-15, and IL-18 have demonstrated the ability to activate NK cells against hepatocellular carcinoma (HCC), melanoma, and breast cancer [[9], [10], [11], [12]]. IL-15 and IL-21 combination can upregulate the proliferation and cytotoxicity of NK cells in solid tumors [13,14]. IL-15 and IL-18-mediated activation of NK cells can enhance IL-27 synergy [15]. Nevertheless, they can't easily infiltrate tumor tissues in solid tumors including liver cancer due to immunosuppressive in tumor microenvironment (TME). Hence, the application of adaptive NK cell transplantation has been limited in patients with solid tumors [16]. Consequently, novel clinical strategies are required to enhance the anti-tumor effects of NK cells in cancer therapy.

In recent studies, there has been considerable interest in the therapeutic applications and potential functions of exosomes in various cancers [17,18]. Understanding the characteristics of exosomes is crucial for exploring their clinical uses. Exosomes, released from various cells are 30–150 nm sizes of small vesicles. They are composed of nucleic acids, proteins, mRNA, lipids, and other biological substances [19] and involved in essential cellular functions such as proliferation and apoptosis [20,21]. Furthermore, they play an important role in immune regulation, in both diseased conditions and healthy states. Exosomes are characterized by specific proteins, including CD9, CD63, CD81, and Alix [19], which serve as exosomal markers for various cells, including immune cells.

The various cells-derived exosomes contain unique proteins, which are exhibited diverse functions based on their cellular origins [22]. Therefore, many studies have explored the potential role of exosomes in various clinical applications, such as drug delivery systems, therapeutic carriers, and biomarkers [[17], [18], [19],^22^]. For instance, exosomes-derived mesenchymal stem cells (MSC-exos) are enriched with bioactive molecules such as growth factors [23], while exosomes-derived dendritic cells (DC-exos) consist of functional MHC class-associated peptides [24]. Exosomes-derived NK cells (NK-exos) are enriched with specific cytotoxic proteins, including granzyme B and perforin, which exhibit tumor-killing abilities against cancers [[25], [26], [27], [28]]. Notably, NK-exos offer various advantages for cancer therapy. The nano-sized NK-exos can easily infiltrate and diffuse tumor tissues. They demonstrate potent therapeutic effects and possess active targeting capabilities. Hence, NK-exos can overcome the limitations of NK cell including immunosuppression in tumor microenvironment (TME) [29]. The cytotoxic activity of extracellular vesicles-derived NK cells (NK-EVs) against breast cancer and glioblastoma can be enhanced by cytokine, IL-15 [30]. Nevertheless, the specific mechanisms and anti-tumor effects of NK-exos stimulated with IL-15 and other cytokines such as IL-21 (NK-exos^IL−15/21^) in solid tumors, including HCC, remain unclear.

This study first reported on the investigation of the therapeutic effects, including cytotoxicity and apoptosis of NK-exos-stimulated with IL-15 and IL-21 in HCC therapy. To determine the novel signaling mechanism underlying of NK-exos^IL−15/21^, we evaluated the role of cytotoxic molecules, as important regulators of cell cytotoxicity and apoptosis in HCC cells. Our results demonstrated that NK-exos^IL−15/21^ significantly increased the cytotoxic and apoptotic activity against Hep3B cells by activating the pro-apoptotic proteins (e.g., perforin, granzyme B, Bax, cleaved caspase 3, and cleaved PARP), and by inhibiting the anti-apoptotic protein, Bcl-2. NK-exos^IL−15/21^ can provide promising strategies for the treatment of solid tumors, especially in HCC.

## 2 Materials and methods

### 2.1 Cell lines and cell culture

Human NK-92 cells purchased from the American Type Culture Collection (ATCC; Manassas, VA, USA, #CRL-2407) were cultured at 3 × 10^5^ cells/mL for 72 h using T-25, T-75, and T-175 flasks in alpha minimum essential medium (α-MEM; Corning Inc., Corning, NY, USA, #10-022-CVR) supplemented with 12.5% horse serum (HS; Sigma-Aldrich, St. Louis, MO, USA, #26050-088), 12.5% fetal bovine serum (FBS; Corning Inc., #35-015-CV), 1% penicillin-streptomycin (P/S; Corning Inc., #30-002-CI), 10 ng/mL IL-2 (Miltenyi Biotec, Bergisch Gladbach, Germany, #130-097-745), 0.2 mM myo-inositol (Sigma-Aldrich, #87-89-8), 0.1 mM 2-mercaptoethanol (Sigma-Aldrich, #M6250), and 0.02 mM folic acid (Sigma-Aldrich, #59-30-3) at 37 °C with 5% CO_2_, according to the manufacturer's instructions. Hep3B cells purchased from the Korean Cell Line Bank (KCLB; Seoul, Korea, #88064) were cultured in Dulbecco's modified Eagle medium (DMEM; Corning Inc., #10-013-CV) supplemented with 10% FBS (Corning Inc., #35-015-CV) and 1% P/S (Corning Inc., #30-002-CI) at 37 °C with 5% CO_2_, following the manufacturer's instructions.

### 2.2 Exosome isolation

NK-92 cells (5 × 10^5^ cells/mL) were cultured with IL-15 (10 ng/mL; Miltenyi Biotec, #130-095-765) and IL-21 (10 ng/mL; Miltenyi Biotec, #130-095-784) for 48 h, supplemented with exosome-depleted HS or FBS, prepared by ultracentrifugation (Himac CP100NX; HITACHI, Tokyo, Japan, #5720110011) at 120,000×*g* for 18 h at 4 °C [31]. The culture medium was first centrifuged at 300×*g* for 5 min, at 2,000×*g* for 15 min, and again the collected supernatant was centrifuged at 10,000×*g* for 30 min. Subsequently, the exosomes containing supernatant were filtered (0.22 μm) and concentrated using a tangential flow filter system (TFF-Easy; HansaBioMed Life Sciences, Tallinn, Estonia) [32]. The exosomes were dissolved in phosphate buffered saline (1 mL PBS; Corning Inc., #21-040-CV).

### 2.3 Exosome identification

To assess the structure of exosomes using transmission electron microscopy (TEM; JEOL, Tokyo, Japan, # JEM-1400), The exosomes were fixed with 2% paraformaldehyde and placed on a Formvar/carbon-coated TEM grid (Agar Scientific, Stansted Mountfitchet, UK, #AGS162). The grid was treated with 0.05 M glycine for 10 min to quench free aldehyde groups and blocked with blocking buffer (PBS containing 1% BSA) for 30min. Then, sequentially exosomes were incubated with specific antibody to CD9 (System Biosciences, Palo Alto, CA, USA, #EXOAB-CD9A-1) for 3 h and with Goat anti-rabbit IgG conjugated to gold particle (Alexa Flour 488, Invitrogen Life Technologies, Grand Island, NY, #A-31566) for 1 h. Next, the grid was washed twice with a small drop of PBS to remove unbound antibodies, and then it was allowed air-dry. Exosomes on grid were visualized using a JEM-1400 TEM operating at 80 kV. Digital images were captured using a Mega-view G3 Camera (EMSIS, Muenster, Germany, #S03U).

The sizes of exosome particles were determined by dynamic light scattering (DLS; Malvern Instrument, Malvern, UK, #Zetasizer Nano ZS) equipped with a 633 nm laser, and also nanoparticle tracking analysis (NTA; Malvern Instrument, Worcestershire, UK, #Nanosight LM10) equipped with a 405 nm laser. The measurements were performed using NTA software (Malvern Instrument, #Nanosight version 3.1).

### 2.4 Exosome uptake assay

Exosomes were labeled with PKH67 (Green Fluorescent Cell Linker Mini Kit; Sigma-Aldrich, #PKH67GL-1 KT) according to the manufacturer's instructions. PKH67-labeled exosomes (50 μg) were incubated with Hep3B cells for 24 h at 37 °C. Aliquots of the cell suspension were placed on microscope slides and mounted with a coverslip using Aqua-Poly/Mount (Polysciences, Inc., Warrington, PA, USA, #18606-20). Nuclei were stained blue using Hoechst 33258 (Sigma-Aldrich, #23491-45-4). The cellular uptake of exosomes was visualized using a confocal laser scanning microscope (CLSM; Carl Zeiss, Oberkochen, Germany, #LSM 880).

### 2.5 Live/dead assay

Hep3B cells co-treated with exosomes were measured using a live/dead assay kit (Thermo Fisher Scientific, #L3224) following the manufacturer's instructions. After 24 h, the cells were washed and incubated in PBS containing 4 μM ethidium homodimer I (red) and 0.5 μM calcein-AM (green) for 30 min. The confocal microscope (Carl Zeiss, #LSM 880) was used to obtain the fluorescence images. Green and red fluorescence signals represent living cells and dead cells, respectively.

### 2.6 Cell viability assay

To assess the viability effects of NK-exos^IL−15/21^ in HCC cells, a 3-(4,5-dimethylthiazol-2-yl)-2,5-diphenyltetrazolium bromide (MTT; Dojindo Molecular Technologies, #298-93-1) assay was conducted following the manufacturer's instructions. Hep3B cells (1 × 10^4^ cells/well) were co-treated with exosomes (50 μg) for 24 h. MTT solution (10 μL) was added to each well and re-incubated for 3 h. Thereafter, the medium was removed, and dimethyl sulfoxide (DMSO, 100 μL) was added. To determine cell viability, the absorbance was then read at 570 nm using a microplate reader (Varioskan Flash, Thermo Fisher Scientific, #0250030).

### 2.7 Apoptosis analysis

The synergistic effects of NK-exos^IL−15/21^ on cell apoptosis were evaluated using an apoptosis detection kit (Sigma-Aldrich, #APOAF) following the manufacturer's instructions. The cell staining was evaluated with Annexin V-fluorescein isothiocyanate (FITC) conjugated antibody (Annexin V-FITC) and propidium iodide (PI) [33]. In brief, Hep3B cells (1 × 10^6^ cells/well) were cultured with exosomes (50 μg) for 24 h. After incubation, the harvested cells were resuspended in 500 μL of Annexin V-FITC-binding buffer. Then, the resuspended cells were stained with Annexin V-FITC (10 μL) and propidium iodide (PI, 10 μL) staining solution for 15 min at room temperature in the dark. The stained cells were analyzed using a magnetic-activated cell sorting flow cytometer (MACSQuant VYB; Miltenyi Biotec, #130-096-116) within 2 h. Forward scatter area (FSC-A) and side scatter area (SSC-A) gates were used to remove debris and dead cells. Additionally, forward scatter height (FSC–H) were used to gate on doublets. The fluorescent-positive cells were gated at excitation/emission wavelengths 525/50 nm for Annexin V-FITC and 614/50 nm for PI, respectively.

### 2.8 Western blot analysis

The proteins from NK-92 cells and isolated exosomes were lysed using RIPA buffer (GenDEPOT, Barker, TX, USA, #20–188) supplemented with 1% protease inhibitor (GenDEPOT). Protein samples were loaded onto 10% SDS-PAGE and transferred onto PVDF membranes (Millipore, Billerica, MA, USA, #IPVH00010). After blocking with 1X TBST (pH 7.4; GenDEPOT, #T8056-100) dissolved in 5% skim milk (Sigma-Aldrich, #M7409) for 1 h at room temperature, exosome transferred membranes were sequentially incubated with specific primary antibodies (Santa Cruz Biotechnology, Dallas, TX, USA, #sc-2357) and horseradish peroxidase-conjugated secondary antibodies (Santa Cruz Biotechnology, #sc-516102) [34]. The protein bands from membrane were visualized using the Amersham^TM^ Imager 600 imaging system (GE Healthcare, Chicago, IL, USA, #29-0834-61), and analyzed protein quantifications using ImageJ software (Ver. 1.53e; NIH, Bethesda, MD, USA). The primary antibodies used in this study included perforin (#sc-373943), granzyme B (#sc-8022), CD63 (#sc-5275), β-actin (#sc-47778), and GAPDH (#sc-97724) from Santa Cruz Biotechnology (Dallas, TX, USA); CD9 (#EXOAB-CD9A-1) from System Biosciences (Palo Alto, CA, USA); PARP (#9532Bcl-2 (#15071), Bax (#5023), and Caspase 3 (#9662) from Cell Signaling Technology (Danvers, MA, USA).

### 2.9 Statistical analysis

All experiments were performed in triplicate. The data were analyzed by Student's t-test and presented as the mean ± standard deviation (SD). Significance levels are presented as N.S. (no significance), *p < 0.05, **p < 0.01, and ***p < 0.001.

## 3 Results

### 3.1 Characterization of NK-exos^IL−15/21^

**Fig. 1:**
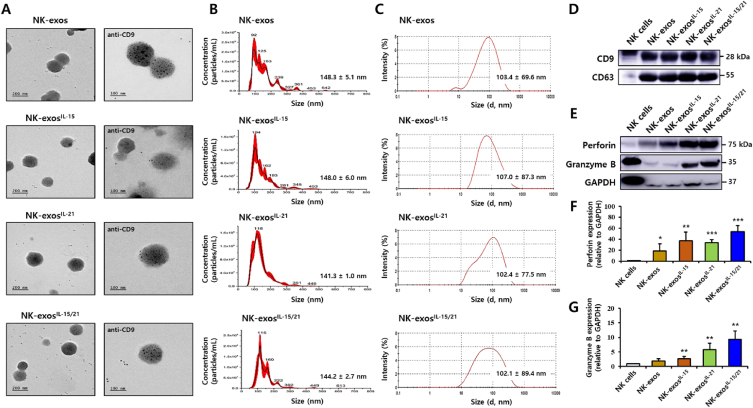
Characterization of NK-exos^IL−15/21^. (A) The morphology of isolated NK-exos, NK-exos^IL−15^, NK-exos^IL−21^, and NK-exos^IL−15/21^ was visualized by immunogold-transmission electron microscopy (TEM) with the antibody for exosomal marker, CD9. Gold particles are depicted as black dots. Scale bars: 200 nm (left) and 100 nm (right). (B, C) Size distribution determined by nanoparticle tracking analysis (NTA; B) and dynamic light scattering (DLS; C). (D, E) Western blotting of the expression levels of exosomal markers, including CD9 and CD63 (D), and cytotoxic proteins, including perforin and granzyme B (E), in comparison with the expression of the negative control marker (GAPDH). Uncropped western blots were indicated in Supplementary Figs. S2 and S3. (F, G) Quantification of protein expression normalized to that of GAPDH. All data are presented as the mean ± SD (*n* = 3). *p < 0.05, **p < 0.01, and ***p < 0.001 vs. NK cells. (For interpretation of the references to colour in this figure legend, the reader is referred to the Web version of this article.)

Many cytokines exhibit a synergistic effect on inducing cytotoxicity and proliferation of NK cells [9,[13], [14], [15],^35^]. To investigate the cytotoxicity, we assessed the effects of different cytokines such as IL-12, IL-15, IL-18, and IL-21, in stimulated with NK-92 cells using western blotting. We observed that the expression levels of cytotoxic proteins (perforin and granzyme B) were significantly elevated in IL-15 and IL-21 stimulated NK-92 cells compared to the other cytokines, as reported in the Supplementary Fig. S1. To further investigate the characteristics of NK-exos stimulated with IL-15 and IL-21 (NK-exos^IL−15/21^), NK-92 cells were co-cultured with IL-15 and IL-21, followed by the isolation of exosomes through ultracentrifugation. Isolated exosomes were examined using transmission electron microscopy (TEM), nanoparticle tracking analysis (NTA), dynamic light scattering (DLS), and Western blot analysis. The morphology of NK-exos, NK-exos^IL−15^, NK-exos^IL−21^, and NK-exos^IL−15/21^ was investigated by immunogold TEM with the antibody for CD9 exosomal marker. The black dots showed the location of the immunogold particles on the surface of exosomes. They were also positive for the CD9 exosomal marker (Fig. 1A). Exosomes are 30–150 nm sizes of small vesicles [19]. The size and number of exosomes were determined using NTA (Fig. 1B) and DLS (Fig. 1C), revealing a range of 100–150 nm. Compared to NK-exos, NK-exos^IL−15^, NK-exos^IL−21^, and NK-exos^IL−15/21^ exhibited similar morphology and size without significant changes. Furthermore, exosomes are characterized by the presence of specific surface markers such as Alix, CD9, and CD63 [19,36]. The isolated NK-exos^IL−15/21^ were found to express specific exosomal markers, including CD9 and CD63 (Fig. 1D and Supplementary Fig. S2). NK-exos have been reported to contain cytotoxic proteins known to induce apoptosis (e.g., perforin and granzyme B) [25,26,37,38] and to exert anti-tumor effects in various cancer cells [[25], [26], [27], [28]]. Notably, western blot analysis revealed the enriched expression of cytotoxic proteins (perforin and granzyme B) in exosomes (Fig. 1E–G and Supplementary Fig. S3). Conversely, the expression of the negative control protein (GAPDH) was found to be lower in exosomes. Overall, these findings provide evidence supporting the effective characterization of NK-exos^IL−15/21^.

### 3.2 Uptake of NK-exos^IL−15/21^ by HCC cells

**Fig. 2:**
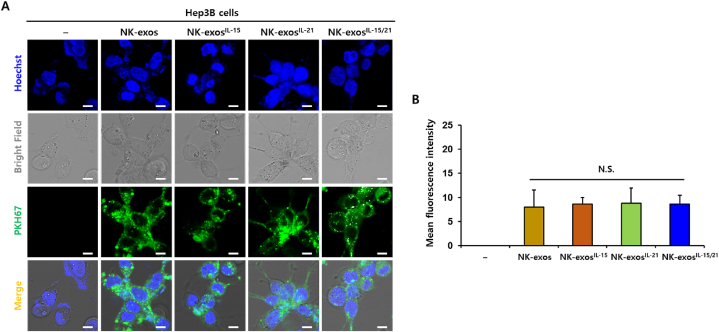
Cellular uptake of NK-exos^IL−15/21^ by HCC cells. (A) Co-culture of Hep3B cells with PKH67-labeled NK-exos, NK-exos^IL−15^, NK-exos^IL−21^, and NK-exos^IL−15/21^ (each 50 μg, green) for 24 h. Internalization was visualized by confocal microscopy. Cell nuclei were stained with Hoechst 33258 (Blue). Scale bars: 20 μm. (B) Uptake levels of NK-exos, NK-exos^IL−15^, NK-exos^IL−21^, and NK-exos^IL−15/21^ by Hep3B cells. All data are presented as the mean ± SD (*n* = 3). *N.S.* (no significance) vs. NK-exos. (For interpretation of the references to colour in this figure legend, the reader is referred to the Web version of this article.)

Exosomes can be internalized into target cells through multiple mechanisms, including receptor-ligand interaction, endocytosis, and membrane fusion [19]. To assess the internalization of NK-exos^IL−15/21^ by HCC cells, Hep3B cells were co-incubated with PKH67-labeled NK-exos, NK-exos^IL−15^, NK-exos^IL−21^, and NK-exos^IL−15/21^ for 24 h. Subsequently, the cellular uptake of NK-exos^IL−15/21^ by Hep3B cells was visualized using a confocal microscopy (Fig. 2A). Moreover, NK-exos^IL−15/21^ showed similar uptake levels to NK-exos, NK-exos^IL−15^, and NK-exos^IL−21^ (Fig. 2B). These findings indicate that NK-exos^IL−15/21^ are internalized into Hep3B cells and localized in the cytoplasm.

### 3.3 Enhancement of cytotoxicity and reduction of cell viability by NK-exos^IL−15/21^ in HCC cells

**Fig. 3:**
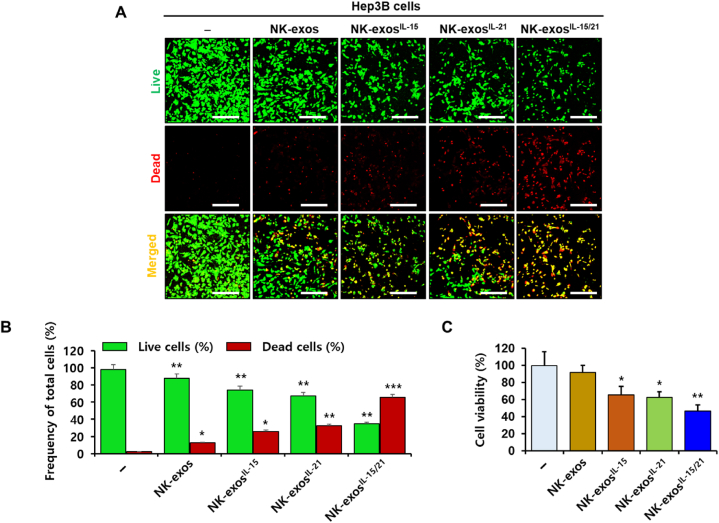
Cytotoxicity and cell viability of NK-exos^IL−15/21^ in HCC cells. Hep3B cells were co-treated with NK-exos, NK-exos^IL−15^, NK-exos^IL−21^, and NK-exos^IL−15/21^ (each, 50 μg) for 24 h. (A) Staining of Hep3B cells with a staining solution containing 0.5 mM calcein-AM and 4 mM EthD-1 for 30 min. Live (green) and dead (red) cells were detected by live/dead assay and visualized by confocal microscopy. Scale bars: 200 μm. (B) Quantification of live and dead cells. (C) Measurement of the cell viability of Hep3B cells by MTT assay. All data are presented as the mean ± SD (*n* = 3). *p < 0.05, **p < 0.01, and ***p < 0.001 vs. untreated Hep3B cells. (For interpretation of the references to colour in this figure legend, the reader is referred to the Web version of this article.)

The cell proliferation and apoptosis of HCC have been reported to regulate via the mechanism of NK cells [39,40]. IL-15, known to major cytokine stimulates the biological function, including cytotoxicity and apoptosis in NK cells [8,10,15,41]. In recent years, many investigations have focused on the potential anti-tumor effects of NK-exos against various cancers (e.g., neuroblastoma, melanoma, breast cancer, and lung cancer) [[25], [26], [27], [28]]. Notably, IL-15 cytokine priming significantly enhanced the anti-tumor efficacy of NK-EVs for glioblastoma treatment [30]. However, the functional mechanisms and signaling pathways of NK-exos, which exhibit a biological activity in cancer therapy, are not fully understood. Thus, it is necessary to investigate the synergistic effects of IL-15 in combination with other cytokines such as IL-21 on the biogenesis of NK-exos. In this study, we examined the effects of NK-exos-stimulated with IL-15 and IL-21 synergy on the cytotoxicity and cell viability of HCC cells. To determine the cytotoxic activity of NK-exos^IL−15/21^ in Hep3B cells, we assessed a live/dead assay. Live (green) and dead (red) cells were visualized using a confocal microscope (Fig. 3A) and quantified (Fig. 3B). NK-exos^IL−15/21^ led to a significant decrease in the number of live cells, whereas an increase in the number of dead cells was observed (Fig. 3B). To further evaluate the effects of NK-exos^IL−15/21^ on the viability of Hep3B cells, we performed an MTT assay. The findings revealed a significant reduction in cell viability upon treatment with NK-exos^IL−15/21^ compared to NK-exos, NK-exos^IL−15^, and NK-exos^IL−21^ (Fig. 3C). Taken together, these findings demonstrate that NK-exos^IL−15/21^ exhibit enhanced cytotoxicity and reduced cell viability in HCC cells.

### 3.4 Improvement of apoptotic activity by NK-exos^IL−15/21^ in HCC cells

**Fig. 4:**
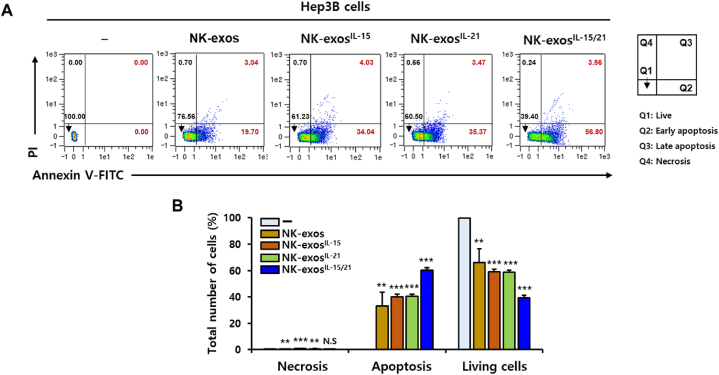
Apoptotic activity of NK-exos^IL−15/21^ in HCC cells. (A) Co-culture of Hep3B cells with NK-exos, NK-exos^IL−15^, NK-exos^IL−21^, and NK-exos^IL−15/21^ (each, 50 μg) for 24 h. The cells were stained with Annexin V-FITC/PI and analyzed by flow cytometry. Q1: live cells (Annexin V−/PI−); Q2: early apoptotic cells (Annexin V+/PI−); Q3: late apoptotic cells (Annexin V+/PI+); Q4: necrotic cells (Annexin V−/PI+). (B) Quantification of necrotic, apoptotic, and live cells. All data are presented as the mean ± SD (*n* = 3). **p < 0.01, ***p < 0.001, and *N.S.* (no significance) vs. untreated Hep3B cells.

As shown in Fig. 3, NK-exos^IL−15/21^ exhibited the strong cytotoxicity and viability reduction in Hep3B cells. Next, we further investigated the apoptotic activity of NK-exos^IL−15/21^ in HCC cells using flow cytometric analysis (Fig. 4A). The viable Hep3B cells (Q1) were significantly decreased, whereas the early and late apoptotic Hep3B cells (Q2+Q3) were significantly increased following exposure to NK-exos^IL−15/21^ (Fig. 4B). Necrosis (Q4) didn't no significant effect on exposure to NK-exos^IL−15/21^. These results suggest that NK-exos^IL−15/21^ induce strong apoptotic effects on HCC cells.

### 3.5 Potential signaling mechanism of NK-exos^IL−15/21^ for apoptosis effect in HCC cells

As shown in Fig. 4, NK-exos^IL−15/21^ enhanced the apoptotic activity of HCC, Hep3B cells. Cells undergo apoptosis through two major pathways, the intrinsic pathway (including mitochondrial pathway) or the extrinsic pathway (including death receptor pathway) [[42], [43], [44], [45], [46], [47], [48], [49]].

**Fig. 5:**
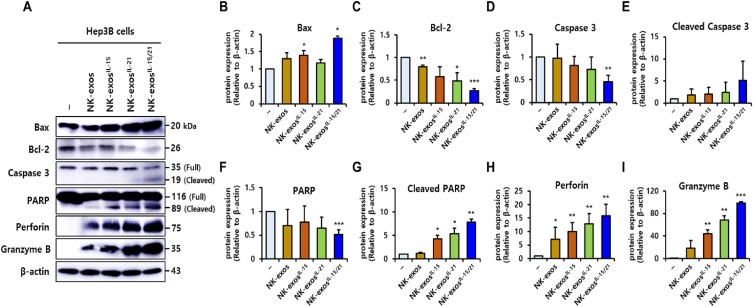
The expression of apoptotic proteins enhanced by NK-exos^IL−15/21^ in HCC cells. (A) Western blotting of the expression levels of specific pro-apoptotic proteins (Bax, caspase 3, PARP, perforin, and granzyme B) and an anti-apoptotic protein (Bcl-2). β-actin was used as a loading control. Uncropped western blots were indicated in Supplementary Fig. S4. (B–I) Quantification of protein expression normalized to that of β-actin. All data are presented as the mean ± SD (*n* = 3). *p < 0.05, **p < 0.01, and ***p < 0.001 vs. untreated Hep3B cells.

First, the intrinsic apoptosis pathway is involved in lytic granules containing granzyme B and perforin, which trigger the cytochrome c release from mitochondria, and then activate caspase signaling to drive cell apoptosis. Second, the extrinsic apoptosis pathway is involved in death ligands (e.g., FasL and TRAIL) bind to the death receptors (e.g., Fas and TRAIL-R) on target cells, activating cytotoxicity and initiating caspase-dependent apoptotic cell death. Here, we investigated the signaling pathway involved in NK-exos^IL−15/21^-mediated apoptosis, with a focus on intrinsic pathway-related cytotoxic proteins (e.g., Bax, Bcl-2, caspases, perforin, and granzyme B). The expression of cytotoxic molecules, which are important regulators of apoptosis, was evaluated using Western blot analysis (Fig. 5A and Supplementary Fig. S4). Western blot analysis of apoptosis provides direct evidence for the presence of specific proteins involved in apoptotic signaling. In Hep3B cells, NK-exos^IL−15/21^ resulted in the upregulation of specific pro-apoptotic proteins (e.g., Bax, cleaved caspase 3, cleaved PARP, perforin, and granzyme B) and inhibited the anti-apoptotic protein Bcl-2, which prevent to cytochrome *c* translocation (Fig. 5B–I). These findings propose that NK-exos^IL−15/21^ upregulate the activation of cytotoxic proteins for apoptosis signaling pathway.

**Fig. 6:**
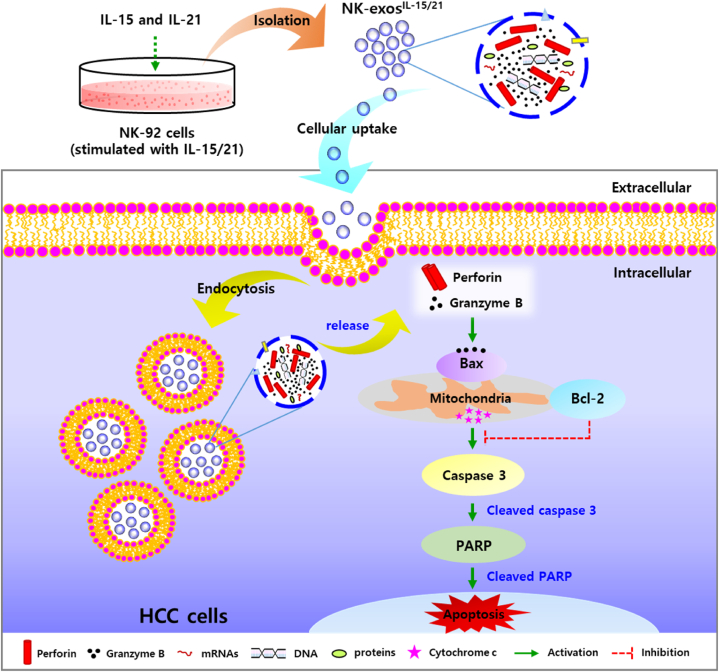
Schematic overview of the apoptosis mechanism for the anti-tumor effects of NK-exos^IL−15/21^ in HCC cells. NK-exos^IL−15/21^ initiate the intrinsic apoptosis pathway by pore-forming protein (perforin)/serine protease (granzyme B) release, and then regulate pro-apoptotic protein, Bax and anti-apoptotic protein, Bcl-2 for the downstream activation of caspase 3. Finally, NK-exos^IL−15/21^ induce apoptosis via PARP activation on HCC cells.

Here, the potential signaling mechanism of NK-exos^IL−15/21^ for HCC cell apoptosis was first demonstrated and summarized as shown in Fig. 6. NK-exos^IL−15/21^ contained more cytotoxic proteins (including perforin and granzyme B) than NK-exos (Fig. 1E–G). NK-exos^IL−15/21^ are taken up into HCC cells by endocytosis, and then release the perforin (pore-forming protein of membrane) and granzyme B (serine protease) for initiating the intrinsic apoptosis pathway. Delivered granzyme B triggers the mitochondrial signaling for apoptosis by interacting with pro-apoptotic Bax protein, inhibiting of anti-apoptotic Bcl-2 protein, and then promote the downstream activation of caspase 3 (namely, cleaved caspase 3) through released cytochrome c (data not shown). Finally, NK-exos^IL−15/21^ induce apoptosis via PARP activation (namely, cleaved PARP) on HCC cells. Taken together, these results provide evidence for a critical role of NK-exos^IL−15/21^ as an apoptosis regulator by enhancing the activation of major cytotoxic molecules in HCC cells.

## 4 Discussion

Recently, there has been increasing interest in exploring the potential applications of nano-sized exosomes in therapeutics. Exosomes derived from different cells are consisted of unique proteins that can modulate their biogenesis. They have shown promise as therapeutic carriers, biomarkers, and vehicles for drug delivery [[17], [18], [19],^22^]. Notably, NK-exos are enriched with cytotoxic proteins, such as perforin and granzyme B, which are derived from NK cells. These two cytotoxic proteins contribute to modulating biological functions such as cytotoxicity and apoptosis against many cancers (e.g., glioblastoma, neuroblastoma, breast cancer, and pancreatic cancer) [30,[50], [51], [52]]. The functional mechanisms of NK-exos increase high tumor cell-killing effects including cytotoxic and apoptotic activity through the cytotoxic proteins, including perforin and granzyme B, trigger the caspases activation-dependent apoptosis pathway in cancers [26,37,50,[53], [54], [55]]. Previously, our study provides the evidence for the strong anti-tumor effects and targeting capability of NK-exos in HCC, at least in part, by novel regulatory mechanisms associated with caspase pathway-dependent apoptosis and the serine/threonine kinase pathway-mediated cell proliferation [56].

Cytokines play an important role in immunotherapeutic approach and synergistic effects when stimulating NK cells, as evidenced by clinical evaluations [[8], [9], [10], [11], [12], [13], [14], [15]]. Nevertheless, there are limitations to the cytokine effects due to their short half-life [57]. Consequently, the application of cytokines in cancer therapy has been limited to only a few cases. In an effort to overcome diverse limitations associated with cytokines, many studies have focused on applying exosomes to enhance the effectiveness of cancer treatment [58,59]. Specifically, NK-EVs primed with IL-15 have demonstrated remarkable cytotoxic activity against various human cancers such as glioblastoma and breast cancer through increasing the expression of apoptotic molecules (e.g., perforin, granzyme B, FasL, and caspases) [30]. Nevertheless, the therapeutic potential of NK-exos in synergy with other cytokines (such as IL-15 and IL-21), has yet to be defined. Thus, studies on cytokines are required to enhance the therapeutic effects of NK-exos.

In this study, we demonstrated the effects of IL-15 and IL-21 cytokines on the functions of NK-exos, highlighting their significant role in inducing potent anti-tumor effects in HCC cells. We characterized NK-exos^IL−15/21^ using various techniques including TEM (Fig. 1A), NTA (Fig. 1B), and DLS (Fig. 1C), confirming their morphology, size, and distribution within the range of 100–150 nm. The effects of IL-15 and IL-21 treated together did not induce a significant change in the size and morphology of NK-exos. Notably, we observed the expression levels of typical exosome markers such as CD9 and CD63 in NK-exos^IL−15/21^ (Fig. 1D). Additionally, the expression levels of perforin and granzyme B, as known to cytotoxic protein, were up-regulated in NK-exos^IL−15/21^ (Fig. 1E–G). NK-exos^IL−15/21^ were taken up by Hep3B cells (Fig. 2A and B). Previous studies have shown that IL-15 and IL-21 have the potential to enhance the proliferation and cytotoxicity of NK cells against solid tumors [13,14]. Interestingly, our findings revealed that the functional effects of NK-exos were potentiated through cytokine stimulation, specifically IL-15 and IL-21. NK-exos^IL−15/21^ exhibited a significant increase in cytotoxicity (Fig. 3A–C) and induced apoptotic activity in Hep3B cells (Fig. 4A and B). These effects were attributed to enhancing the activation of pro-apoptotic proteins (e.g., Bax, cleaved caspase 3, cleaved PARP, perforin, and granzyme B) and the inhibition of an anti-apoptotic protein (Bcl-2) (Fig. 5A–I). Based on the results above, it is reasonable to suggest that co-stimulated IL-15 and IL-21 can affect the characteristics and therapeutic effects on the cytotoxicity and apoptosis of NK-exos. The differential biogenesis of NK-exos^IL−15/^^21^ compared to NK-exos may be attributed to different expression levels of specific molecules associated with cytotoxic and apoptotic functions. These differences in molecular expression may contribute to the enhanced therapeutic effects NK-exos^IL−15/^^21^ in our study. Notably, the enhanced cytotoxicity of NK-exos^IL−15/^^21^ can be attributed to the increased expression and activation of specific cytotoxic proteins, including perforin, granzyme B, and caspases. These findings highlight the crucial role of cytokine stimulation in promoting the cytotoxic ability of NK-exos. The functional significance of these proteins in NK-exos^IL−15/^^21^ is particularly interesting, as their roles in cytotoxic activity against target cells have not been previously reported. These highlights emphasize the novelty of our findings, specifically the potential therapeutic implications of the cytotoxic activity of NK-exos in combination with IL-15 and IL-21 stimulation. Therefore, additional investigations are required to identify the specific proteins that play a significant role in enhancing the biological functions of NK-exos.

The precise mechanisms of HCC apoptosis induced by NK-exos^IL−15/21^ remain poorly understood (Supplementary Table S1). In the present study, these findings provide novel insights into the signaling pathway of apoptosis (notably, intrinsic pathway) of NK-exos^IL−15/21^ in HCC cells and summarized as shown in Fig. 6. NK-exos^IL−15/21^ induce the activation of the intrinsic apoptosis pathway through the release of perforin/granzyme B, resulting in apoptotic cell death, and then modulate pro-apoptotic protein, Bax and anti-apoptotic protein, Bcl-2 for the downstream activation of caspase 3. Finally, PARP activation induce the apoptosis effect on HCC cells. Taken together, these studies are the first report to identify the mechanisms of immunotherapy using NK-exos^IL−15/21^, which can exhibit potential synergistic effects and enhance the anti-tumor immune response.

Further studies focusing on cytokines such as IL-15 and IL-21 will contribute to understanding the regulatory mechanisms of NK-exos in HCC treatment. *In vivo* anti-tumor effects and other apoptotic mechanism (namely extrinsic pathway)-mediated by death ligands (e.g., FasL and TRAIL) are essential to elucidate the potential role of NK-exos^IL−15/21^ in HCC. In the previous report, NK cells exhibit anti-tumor effects through the activation of other immune cells [60]. It would be interesting to further investigation whether NK-exos^IL−15/21^ can enhance the activity of various immune cells (e.g., T cells, B cells, and dendritic cells), leading to a more robust immune response against tumors. Furthermore, NK-exos transfer microRNAs (miRNAs) that regulate gene expression in tumor cells, leading to changes in their behavior and survival [61,62]. NK-exos miR-3607-3p inhibits pancreatic cancer progression by targeting IL-26 [51]. The potential function of NK-exos^IL−15/21^-mediated transfer of miRNAs will be important to better understand the mechanisms underlying the improved anti-tumor immune response.

## 5 Conclusions

Our research findings indicate that NK-exos^IL−15/21^ can enhance the activation of crucial cytotoxic molecules involved in the apoptosis pathway, thereby improving the anti-tumor effects of NK-exos in HCC cells. Based on these results, NK-exos^IL−15/21^ can provide novel insights for the therapeutic strategy of liver cancer, specifically HCC.

## Author contribution statement

In-Young Kim; You Hee Choi: Conceived and designed the experiments; Performed the experiments; Analyzed and interpreted the data; Contributed reagents, materials, analysis tools or data; Wrote the paper.

Ho Yong Kim; Hyeong-woo Song: Performed the experiments; Analyzed and interpreted the data; Contributed reagents, materials, analysis tools or data.

Jong-Oh Park: Conceived and designed the experiments; Contributed reagents, materials, analysis tools or data.

Eunpyo Choi: Conceived and designed the experiments; Analyzed and interpreted the data; Contributed reagents, materials, analysis tools or data; Wrote the paper.

## Data availability statement

Data will be made available on request.

## Funding statement

This work was supported by the 10.13039/501100003725National Research Foundation of Korea (NRF) grant funded by the Korean government (MSIP) to YHC (NRF-2020R1A2C1099712). Moreover, this work was supported by National Research Foundation of Korea (NRF) grant funded by the Korea government (MSIT) (No. 2020R1A5A8018367).

## Declaration of competing interest

The authors declare that they have no known competing financial interests or personal relationships that could have appeared to influence the work reported in this paper.
