# Supplementary material for: Functional enhancement of exosomes derived from NK cells by IL-15 and IL-21 synergy against hepatocellular carcinoma cells: The cytotoxicity and apoptosis in vitro study
Source: Heliyon. 2023 Jun 2;9(6):e16962. doi: 10.1016/j.heliyon.2023.e16962 (PMC10361042; doi:10.1016/j.heliyon.2023.e16962)
Supplement: Multimedia component 1 [file mmc1.pdf]

Supporting information

Functional enhancement of exosomes derived from NK cells by IL-15 and IL-21 synergy against hepatocellular carcinoma cells: The cytotoxicity and apoptosis in vitro study

In-Young Kim[1], Ho Yong Kim[1], Hyeong-woo Song[1], Jong-Oh Park[1,#], You Hee Choi[1,#], Eunpyo Choi[1,2,#]

[1]Korea Institute of Medical Microrobotics, 43-26 Cheomdangwagi-ro, Buk-gu, Gwangju 61011, Republic of Korea

[2]School of Mechanical Engineering, Chonnam National University, 77 Yongbong-ro, Buk-gu, Gwangju 61186, Republic of Korea

#Corresponding authors:

J.-O.P. (jop@kimiro.re.kr), Y.H.C. (youheechoi@kimiro.re.kr),

E.C. (eunpyochoi@jnu.ac.kr)

Supplementary Figures

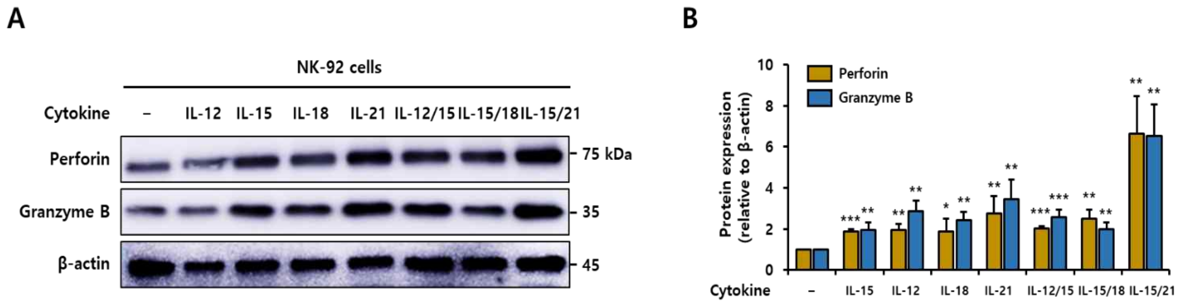

Fig. S1. The cytotoxic protein levels of NK-92 cells stimulated with IL-15 and IL-21. (A) NK-92 cells were cultured alone or in combination with various cytokines, including IL-12, IL-15, IL-18, and IL-21 (each, 10 ng/ml) for 24 h. The expressions of specific cytotoxic proteins were analyzed using western blotting. β-actin was used as a loading control. (B) The quantification of protein expression was normalized to that of β-actin. All data are shown as mean ± SD (n = 3). $^{*}p < 0.05$, $^{**}p < 0.01$, and $^{***}p < 0.001$ vs. the untreated NK-92 cells.

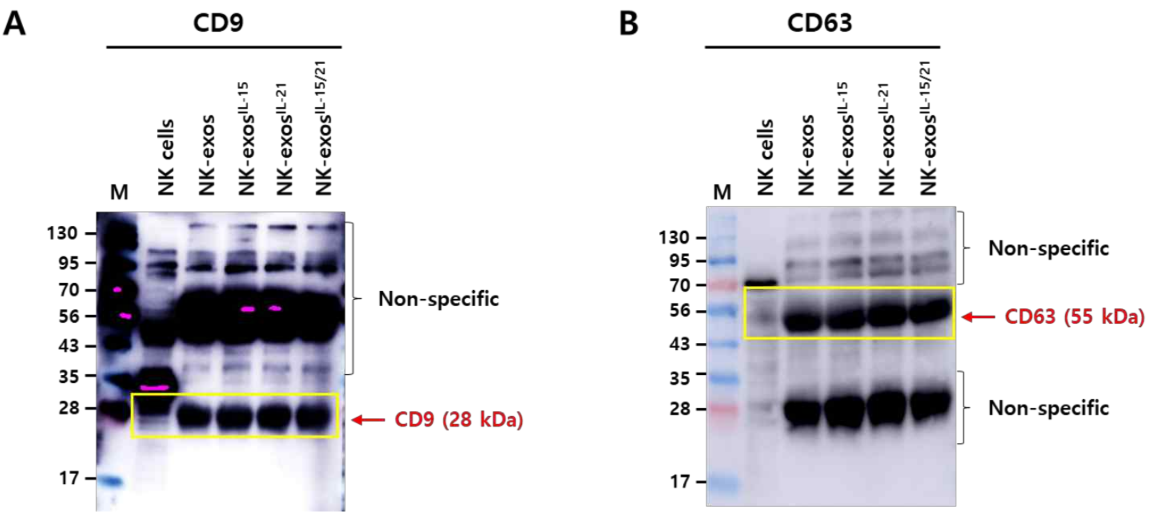

Fig. S2. Western blot full band of exosomal markers. (A, B) The expression levels of exosomal markers, CD9 (A) and CD63 (B). Specific expression band was indicated the yellow box and red arrow. M; protein marker.

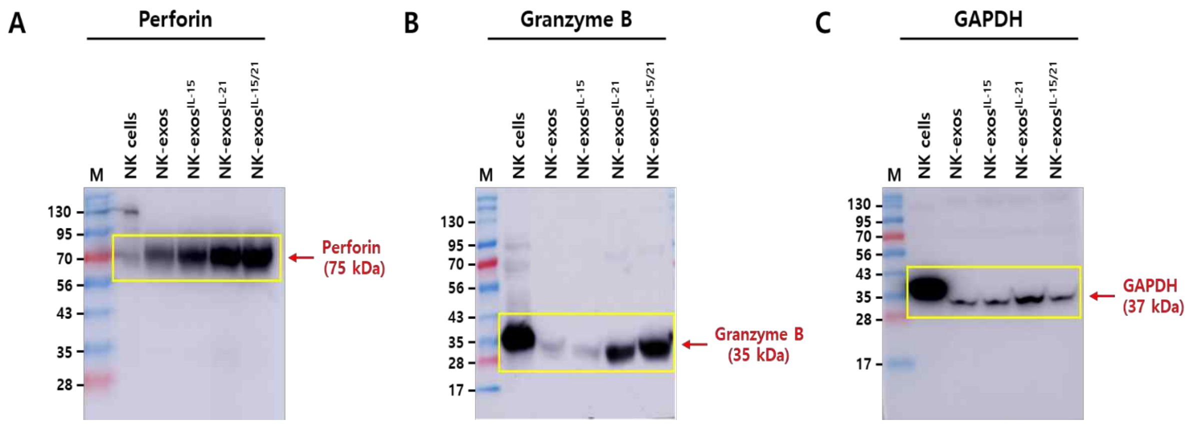

Fig. S3. Western blot full band of cytotoxic proteins and negative control marker. (A, B) The expression levels of cytotoxic proteins, (A) perforin and (B) granzyme B. (C) GAPDH expression is a negative control marker. Specific expression band was indicated the yellow box and red arrow. M; protein marker.

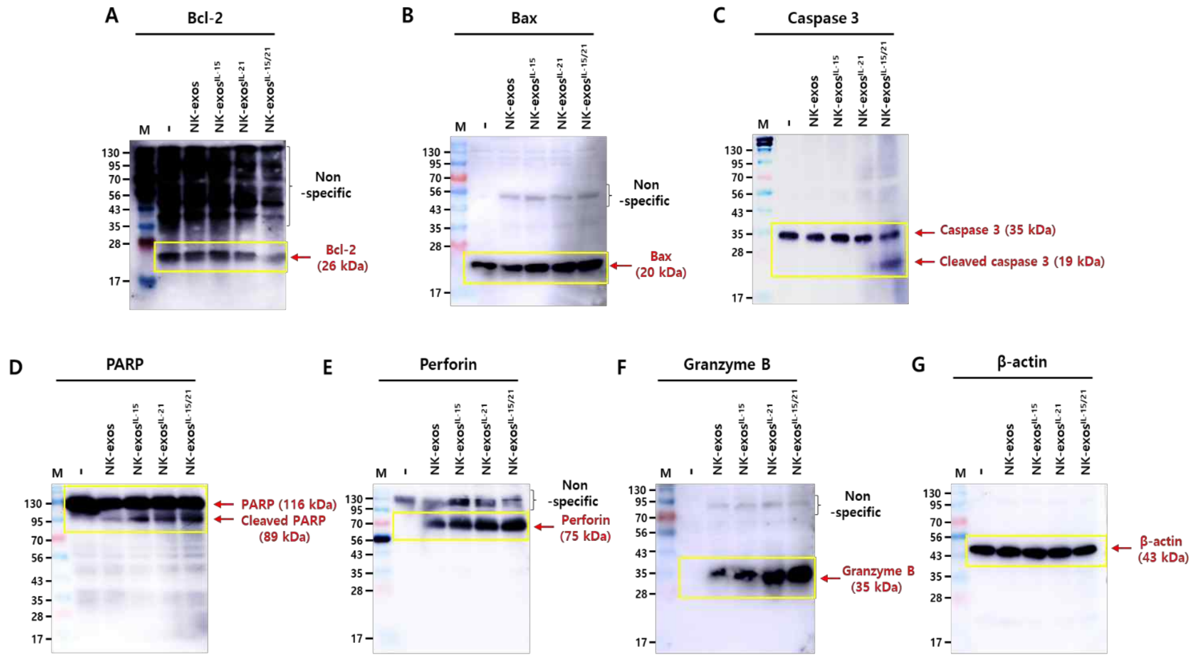

Fig. S4. Western blot full band of anti-apoptotic and pro-apoptotic protein effects in Hep3B cells. (A) The expression levels of anti-apoptotic protein, Bcl-2. (B-F) The expression levels of pro-apoptotic proteins, Bax (B), caspase 3 (C), PARP (D), perforin (E), and granzyme B (F). (G) β-actin expression is a loading control marker. Specific expression band was indicated the yellow box and red arrow. M; protein marker.

NK cell	Cytokine	Target cancer	Cell line	Potential biological effect			Reference
				Biomolecule	Signaling pathway	Function	
NK-92 MI	IL-15	Glioblastoma<br>Breast cancer<br>Thyroid cancer	U87/MG<br>MDA-MB-231<br>CAL-62	Perforin<br>Granzyme B<br>Fas L<br>Caspase<br>Bax, Bcl-2	Activate intrinsic and extrinsic apoptosis	Enhance cytotoxicity and apoptosis<br>Inhibit tumor growth	[1]
PBMC	IL-2<br>IL-15	Leukemia	K562<br>NALM-18	DNAM1 (CD226)<br>DNAM1-L<br>(CD155/CD112)<br>Caspase	Activate extrinsic apoptosis	Enhance cytotoxicity and apoptosis	[2]
PBMC<br>or<br>NK-92	IL-2<br>IL-12<br>IL-15<br>IL-18	Colorectal carcinoma<br>Prostate carcinoma<br>Breast adenocarcinoma<br>Gland ductal carcinoma<br>Ovarian adenocarcinoma<br>Metastatic melanoma<br>Glioblastoma	HCT116<br>DU145<br>SK-BR-3<br>T-4D7<br>OVCAR-3<br>WM9<br>U87/MG	NKG2D<br>Perforin<br>Granzyme B<br>DNAM1 (CD226)<br>DNAM1-L<br>(CD155/CD112)<br>Caspase	Activate intrinsic and extrinsic apoptosis	Enhance cytotoxicity and apoptosis	[3]

Table S1. Signaling mechanisms and biological effects of NK-exos stimulated with various cytokines. PBMC, peripheral blood mononuclear cell; NK-92MI, interleukin-2-independent cell line derived from natural killer-92; IL-2, interleukin-2; IL-12, interleukin-12; IL-15, interleukin-15; IL-18, interleukin-18; NKG2D, natural killer group 2 member D; Fas L, Fas ligand; DNAM1, DNAX accessory molecule 1; DNAM1-L, DNAX accessory molecule 1-ligand.

Supplementary Table S1 References

[1] Zhu L, et al. Enhancement of antitumor potency of extracellular vesicles derived from natural killer cells by IL-15 priming. Biomaterials. 2019; 190, 38-50.

[2] Di Pace AL, et al. Characterization of Human NK Cell-Derived Exosomes: Role of DNAM1 Receptor In Exosome-Mediated Cytotoxicity Against Tumor. Cancers (Basel). 2020; 12, 661.

[3] Aarsund M, et al. Comparison of characteristics and tumor targeting properties of extracellular vesicles derived from primary NK cells or NK-cell lines stimulated with IL-15 or IL-12/15/18. Cancer Immunol. Immunother. 2022; 71, 2227-2238.
